# Improving spatial data in health geographics: a practical approach for testing data to measure children’s physical activity and food environments using Google Street View

**DOI:** 10.1186/s12942-021-00288-8

**Published:** 2021-08-18

**Authors:** Jesse Whitehead, Melody Smith, Yvonne Anderson, Yijun Zhang, Stephanie Wu, Shreya Maharaj, Niamh Donnellan

**Affiliations:** 1 School of Nursing, University of Auckland, Private Bag 920019, Auckland, 1142 New Zealand; 2 Department of Paediatrics, Child and Youth Health, University of Auckland, Level 1, Building 507, Grafton Campus, Private Bag 92019, Auckland, 1142 New Zealand; 3 Department of Paediatrics, Taranaki Base Hospital, Taranaki District Health Board, David Street, New Plymouth, 4310 New Zealand; 4 Tamariki Pakari Child Health and Wellbeing Trust, Taranaki, New Zealand; 5 Faculty of Health and Medical Sciences, University of Auckland, Private Bag 920019, Auckland, 1142 New Zealand

**Keywords:** Measurement, Neighbourhood environments, Child health, Health behaviours, Health geography, Child-friendly cities

## Abstract

**Background:**

Geographic information systems (GIS) are often used to examine the association between both physical activity and nutrition environments, and children’s health. It is often assumed that geospatial datasets are accurate and complete. Furthermore, GIS datasets regularly lack metadata on the temporal specificity. Data is usually provided ‘as is’, and therefore may be unsuitable for retrospective or longitudinal studies of health outcomes. In this paper we outline a practical approach to both fill gaps in geospatial datasets, and to test their temporal validity. This approach is applied to both district council and open-source datasets in the Taranaki region of Aotearoa New Zealand.

**Methods:**

We used the ‘streetview’ python script to download historic Google Street View (GSV) images taken between 2012 and 2016 across specific locations in the Taranaki region. Images were reviewed and relevant features were incorporated into GIS datasets.

**Results:**

A total of 5166 coordinates with environmental features missing from council datasets were identified. The temporal validity of 402 (49%) environmental features was able to be confirmed from council dataset considered to be ‘complete’. A total of 664 (55%) food outlets were identified and temporally validated.

**Conclusions:**

Our research indicates that geospatial datasets are not always complete or temporally valid. We have outlined an approach to test the sensitivity and specificity of GIS datasets using GSV images. A substantial number of features were identified, highlighting the limitations of many GIS datasets.

## Introduction

Neighbourhood design can have a substantial and pervasive influence on child health [1, 2]. A considerable body of literature demonstrates connections between children’s physical activity and environmental features [3]. Specifically, children’s physical activity is facilitated through residing in neighbourhoods that have higher levels of street connectivity, have adequate infrastructure for safe walking and cycling, have higher population densities, and have higher densities of places of importance (e.g., schools, parks) [4–8].

Environments that promote physical activity may also reduce the risk of obesity in children, although the evidence is less clear [6, 7]. Inconsistent findings exist regarding nutrition environments and child health [3, 8, 9], in part due to heterogeneous methods for defining the environment of interest, and in the environmental variables explored [10]. Inconsistent reporting of research methods further hinders a clear understanding of the extant evidence, leading to calls for improved reporting [11]. Residing in areas where access to, and availability of unhealthy food options such as proximity to fast-food restaurants has been *inversely* related to risk of obesity in children [12]. Conversely, the volume of fast food restaurants (but not proximity to the closest) was associated with higher risk of obesity in a study with over 30,000 youth in the United Kingdom [13]. One study in Aotearoa New Zealand (henceforth referred to as Aotearoa) revealed no direct relationship between unhealthy food environments and unhealthy dietary behaviours or excess body size using structural equation modelling [14]. Instead, greater neighbourhood deprivation and unhealthy dietary behaviours were both significantly related to having a higher waist-to-height ratio, which has been shown to be a useful predictor of metabolic syndrome in children [15].

Socio-economic factors play a key role in understanding environment-health relationships, particularly when considering nutrition environments. Socio-economic inequities in food retail environments have been observed, alongside clustering of food outlets around schools [16–18]. Some evidence exists for gender differences in relationships between nutrition environments and child obesity; Chen and Wang [19] demonstrated significant relationships for girls but not for boys.

### Measurement and indices of children’s health-promoting environments using geographic information systems

Calls have been made to improve sensitivity and specificity in measurement of neighbourhoods and improved built environment metrics for understanding health-environment relationships [20, 21]. A range of child-specific approaches using geographic information systems (GIS) have been developed, including using kernel density to calculate a multicomponent measure of obesogenic environments [22], as well as a measure of school-specific walkability [23] and a child-specific destination accessibility index [24], both calculated within defined buffers. Calculating variables within Euclidean or network buffers around residential and/or school addresses have been the dominant approach in children’s geographies literature [10]. Optimal buffer distances are yet to be determined, with some suggesting between 800 and 1000 m as best for understanding physical activity participation [10, 25]. Alternative approaches to defining neighbourhoods have included kernel density, using participant-drawn or global positioning systems (GPS)-derived “activity spaces” or routes, and administrative boundaries. Each method has its own strengths and weaknesses, and no optimal approach has been determined to date [10].

GIS allows researchers to integrate different datasets with spatial information in powerful ways that are becoming increasingly important for health research and policy [26]. However, additional challenges exist with using GIS to measure environments, including the reliance on access to data that is of sufficient quality and is complete. Across larger regions data may not be available for all areas, especially for different administrative areas where different organisations are responsible for collecting and maintaining data. Furthermore, even if data are complete and accessible, temporal information (i.e., the date that data on built environment features were collected) is often missing. Both the spatial and temporal resolution of GIS data is becoming increasingly important [27], and temporal accuracy, consistency, and validity are considered to be key quality measures [28]. Temporally integrated geographies have the potential to shed new insight into environmental exposure research [29]. Environments, and the GIS data that represents these environments, change over time and therefore the development of ‘temporal’ GIS datasets is a key challenge [30, 31]. Temporally inaccurate data sources, such as GIS datasets of footpaths that are out-of-date, can introduce inaccuracies into analyses by misrepresenting built environments [32]. This is particularly important when the research aim is to assess the impact of exposure to an environment on a specific cohort, and the temporal specificity of outcome data is not contemporaneous with environmental data. Therefore, a core component of ‘temporal’ GIS data is that they seek to monitor and understand environmental changes over time, through quality control information, rather than just reproducing ‘snap-shots’ [30]. A further issue is that datasets may not include all features and locations of importance such as marae (sacred place used by Māori for cultural, religious, and social purposes in Aotearoa) or sites of significance. GIS datasets are often provided ‘as-is’ with little metadata. Moreover, the historical accuracy of data is often unknown, making longitudinal analysis difficult or impossible. Ultimately this can lead to incomplete or inconsistent data both in terms of spatial variables and temporality. The quality of GIS analysis and research depends on the quality of the data used. However, researchers often work with the GIS data that is available, rather than what is most suitable for their research question, leaving potential for the misuse of spatial data [33].

### Spatial metrics to estimate environmental exposure

Determining an appropriate geographic area to estimate environmental exposure is key in studies examining contextual determinants of health [34]. This can involve using ‘static’ concepts of place by examining the administrative units that children live within, based on an assumption that neighbourhood of residence has the most important impact on outcomes of interest, and that individuals living in the same area unit experience the same exposures and contextual effects [34]. However, Kwan et al. [35] argue that in fact, the “most important determinants of exposure are where and how much time people spend while engaged in their daily activities”. These ‘activity spaces’ are the local areas and environments that children travel within and interact with daily, and contain locations children usually visit [34, 36, 37]. Accurately estimating activity spaces can be difficult. The uncertain geographic context problem highlights that there is uncertainty in the spatial extent of individuals experienced environments, as well as temporal uncertainty in the timing and duration of these experiences [34]. Without using GPS trackers to follow the movements of each child across the study period, and temporally weighting locations according to the time spent at them [38], it is difficult to know whether estimated neighbourhoods are a true reflection of the environments that children are exposed to in their daily lives. Selective daily activity bias is another issue. If activity spaces are developed based on locations that people visit, then the features at these locations shape the estimated environment that people are exposed to. The observation of a ‘healthy environment’ may actually reflect individual choices to visit healthy locations [39].

When defining neighbourhood boundaries for individual exposure Jia et al. [40] recommend ensuring that environmental exposure is considered at multiple localities such as the home and school. Research from Aotearoa indicates that the food environments around children’s homes and their schools differs substantially. Fast-food outlets and convenience stores are significantly clustered around both primary and secondary schools [18]. This suggests that these environments should be considered together in order to accurately assess the environments that children are exposed to on a regular basis. Individualised activity spaces can be created for multiple localities by combining home, school, and daily transport environments with a 200 m buffer around the shortest path between each participant’s residence and school [41].

Determining the appropriate metric for an activity space is important as using different approaches to defining neighbourhoods and activity spaces can produce different results [25]. A recent systematic review [10] of GIS-based approaches to measure children’s neighbourhood geographies, mostly conducted in the USA, found that while no singular approach is optimal, studies usually used Euclidean or network buffers ranging from 100 m to 5 km. In the Aotearoa context, a range of different road network buffer sizes from 250 m to 1 km have been used to estimate the extent of children’s neighbourhoods [4, 16, 18, 24, 42]. While adults have different mobility patterns to children, Mavoa and colleagues [25] have comprehensively assessed the impact of buffer size, and suggest that while there is no singular ideal neighbourhood definition, 800 m and 1 km road network buffers produced the most consistent association between the built environment and physical activity in adults.

### Using Google Street View (GSV) to measure environments

Google Street View (GSV) has the potential to act as a rich source of data relating to the built environment in public spaces [43]. While some aspects of GSV imagery have been critiqued, including its patchy global coverage [44], variable collection frequency [45], and variations in capture dates within neighbourhoods [46], the use of GSV has spanned health applications [47], travel patterns [48], and streetscape audits [24, 49]. Tools have been developed to guide the auditing of neighbourhood obesogenic environments using screenshots of GSV images [50]. Importantly, GSV is an emerging historical dataset that can enable the retrospective assessment of environmental variables over time [48, 51], and be used to assess the temporal validity of alternative data sources (e.g., GIS databases). Screenshots of the GSV ‘Time Machine’ function have been captured to examine cross-sectional change in the food environment of the Bronx, New York [52].

In children’s health geographies research, GSV has been used to measure obesogenic advertising in children’s neighbourhoods [53] and on bus stops around schools [54]. This growing area of research has demonstrated the utility of GSV to measure environmental features in relation to health outcomes. As well as being cost effective, GSV holds much potential to fill gaps in missing data (e.g., footpath data which can be missing in GIS datasets [55]). Recently, researchers [56] have demonstrated that batches of GSV images can be downloaded through the Google’s Street View Static API (Application Programming Interface), improving the efficiency of the approach. This approach has also been combined with ‘computer vision’ technology to audit all intersections across an entire country [57], detect and map traffic signs [58], and examine land use [59].

To date, exploring how data from GSV can be triangulated to both validate the historical accuracy of secondary GIS data, and to fill gaps in incomplete GIS datasets has not been explicitly undertaken. Exploring the potential for using historical GSV data to simultaneously measure children’s nutrition and physical activity environments is justified. Therefore, this study aims to develop an approach that tests and improves the sensitivity and specificity of GIS datasets using GSV that can be applied to both children’s nutrition and physical activity environments. We aim to show how GSV can supplement secondary GIS datasets to overcome some of the limitations of these datasets, including the unknown temporal or spatial accuracy of features. This paper adds a novel approach to gap-fill and validate the temporal accuracy of secondary GIS data at the individual activity space level for a study cohort. This builds on previous work in the area by outlining a practical approach for automating the downloading of historic GSV images (rather than relying on manual screenshots from the desktop ‘Time Machine’). It describes an approach and rationale to efficiently access historic GSV images that would allow a detailed assessment of an individual’s entire estimated activity space, including a comprehensive range of features from both the food and physical activity environments that are of particular relevance to childhood obesity. While computer vision and deep learning models are not a specific focus of this paper, we also discuss their potential application to our approach, and how they may further improve the efficiency of GIS data validation, virtual environmental audits, and the replicability of our outlined methodology.

### Setting

**Fig. 1:**
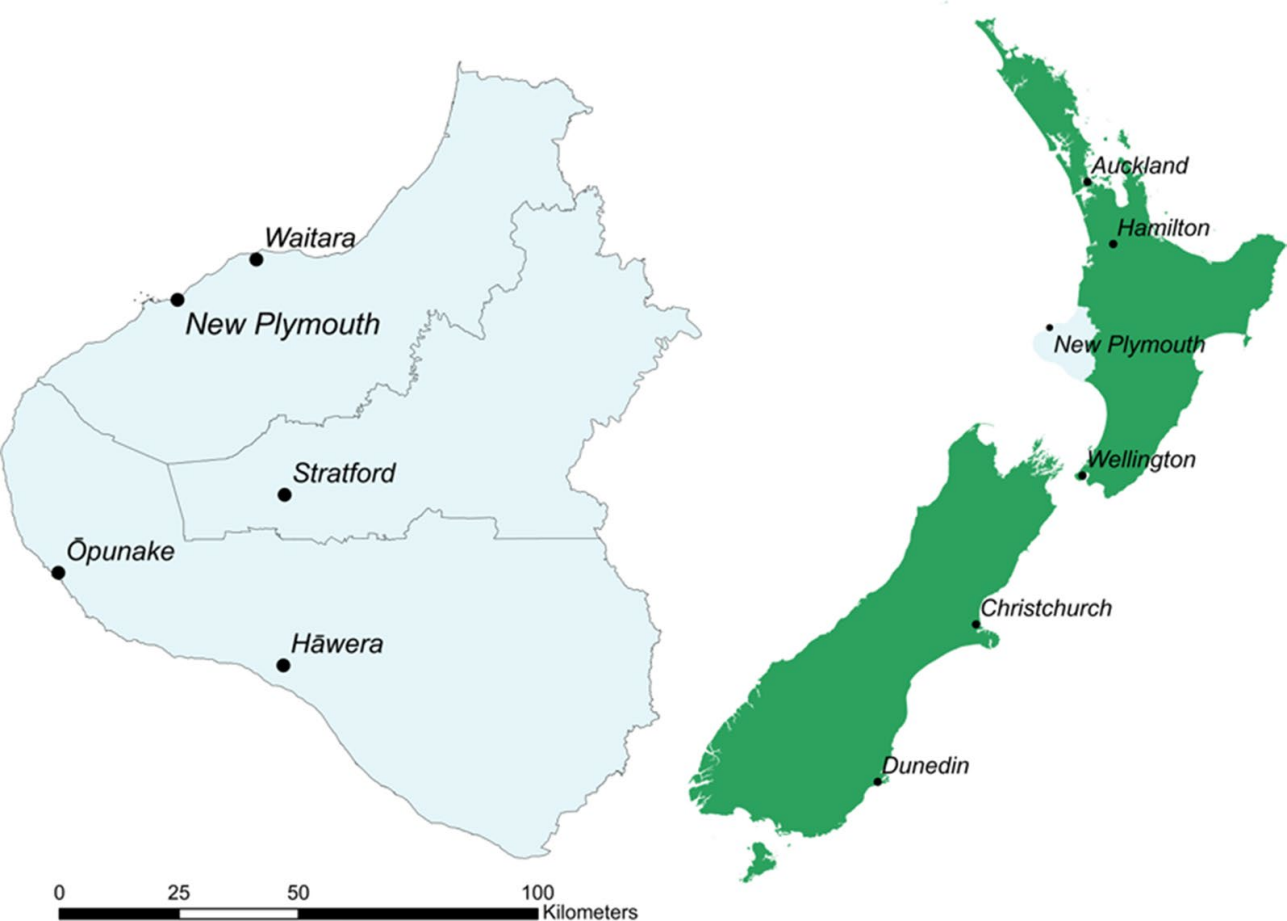
The study area

It is important that any approach to measuring nutrition and physical activity environments is context-specific and relevant. Ideally approaches can also be used across geographic contexts to enable consistency in reporting and understanding health-environment relationships. This research was undertaken with data from Taranaki, Aotearoa, a mixed urban–rural population of approximately 24,688 children aged 0–15 years, of whom 19.8% identify as Māori (Aotearoa’s Indigenous population) [60]. The Taranaki region has historically experienced high levels of childhood obesity [61], therefore, this region was prioritised as a potential geographic setting for this research. Furthermore, while most GSV research in children’s health geographies appears to be conducted in urban settings, it must also be relevant to rural areas where infrastructure and environmental features can differ significantly. The availability and quality of GIS datasets may also differ between rural and urban areas. Therefore, the approach to supplementing and triangulating GIS datasets with GSV data developed in this paper needs to be appropriate to both rural and urban regions with GSV coverage. The city of New Plymouth has around 84,400 residents [62], accounting for 69% of the Taranaki population, and is essentially urban. On the other hand, other areas of Taranaki such as towns in Stratford and South Taranaki districts are much smaller rural towns with different nutritional and physical activity environments, and levels of infrastructure. The urban/rural make-up of Taranaki makes the region an optimal setting to undertake this study. Figure 1 indicates the district council boundaries in the Taranaki region. New Plymouth district is to the north, and includes New Plymouth and Waitara, Stratford district includes the town of Stratford, while South Taranaki district includes Hāwera and Ōpunake.

## Methods

### Study context and overview

This paper is part of a wider study called the Knowing Your Neighbourhood Study (KYNS), which has been designed to develop novel geospatial methods for examining the physical activity and nutritional environments of children. The KYNS adds to a series of studies associated with Whānau Pakari, a multidisciplinary assessment and intervention programme for children and adolescents affected by obesity in Taranaki [63]. Whānau Pakari aims to provide a community-based, family-focused approach to providing support for healthy lifestyle change. A randomised clinical trial was embedded within the programme, and is reported elsewhere [64, 65]. From 2012 to 2016, baseline, 6-month, 12-month, 24-month data were collected, including medical information such as body mass index (BMI) standard deviation score (SDS), dietary behaviour, and physical activity [63]. This programme aimed to reach and engage with those most affected by obesity in the region, namely Māori, and those living in the areas of highest socioeconomic deprivation. This was achieved by ensuring appropriate and acceptable service provision [64]. Baseline data from the Whānau Pakari cohort (98% with a BMI ≥ 98th percentile, 2% with a BMI 91st–98th percentile with weight-related comorbidities at entry) found a higher prevalence of suboptimal dietary behaviours and significant differences in dietary intake when compared with national counterparts [66]. Low levels of physical activity were identified, with the vast majority not meeting national physical activity recommendations [67]. The KYNS will utilise data from Whānau Pakari to examine associations between children’s neighbourhoods and outcomes such as children's physical activity and eating behaviours, as well as BMI SDS.

The aim of the current paper is to outline an innovative approach to testing the temporal accuracy of secondary data. This approach will help to identify any issues with council GIS data provided for the KYNS study, and will supplement these datasets. The final datasets developed in this paper will be used in future KYNS research to develop measures of children’s physical activity and nutrition environments.

**Fig. 2:**
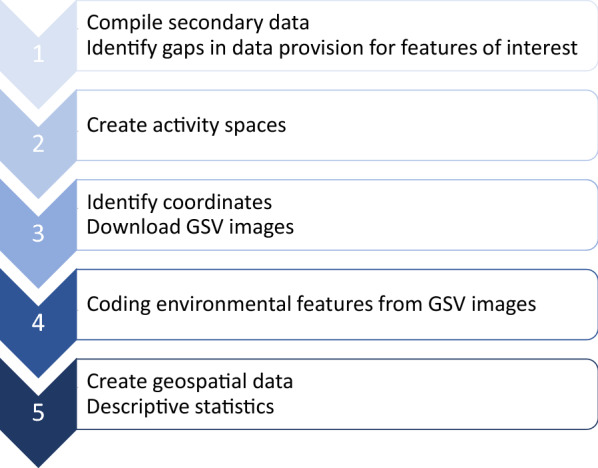
Key steps in methods

Several steps were involved in this approach, which are displayed below in Fig. 2*.* Each stage is also described in further detail in following subsections of this paper.

### Stage 1: data sources and availability

The overarching aim of the KYNS is to develop an index of healthy environments of children, and to utilise it to assess the impact the physical activity and food environment has on children’s health and both dietary and physical activity behaviours. Jia [68] has recently led a series of systematic reviews and meta-analyses of child-specific obesogenic environmental studies, and outlined 10 key spatial indicators for built environments, six spatial indicators of food environments, and two other groups of factors—natural environmental and traffic-related. Jia’s research was used to inform the environmental variables that could be included in a novel index, and several potential data sources were identified. These included open-source (usually Government) datasets and data provided by district councils in the study area. Table 1 defines the data sources compiled for this study, how many different datasets are in each group, and the distribution of this data across the study region.

**Table 1:** Data sources used to develop the Knowing Your Neighbourhood Study dataset, by region and district

Type of source	Taranaki region total (*n*)	New Plymouth District (*n*)	Stratford District (*n*)	South Taranaki District (*n*)
Eligible children in Whānau Pakari	136	95	6	35
Open-source data	5	5	5	5
District council data	11	10	7	6

The residential addresses of eligible Whānau Pakari participants were geocoded and utilised to define the spatial extent of the study area. Five relevant open-source datasets were accessed, all of which were either national or regional datasets, and therefore had the same level of coverage for the entire study region. Table 2 outlines each open-source dataset that was downloaded, its source, and its use in the study. While many open-source datasets provide important information, most of the detailed built environment data used in this research was provided by district councils in the Taranaki region.^1^

**Table 2:** Open-source data accessed for the Knowing Your Neighbourhood Study

Data	Source	Use
Taranaki region schools	Education Counts [69]	Creating activity spaces; Defining the study area and study regions
NZ Road centrelines	Land Information New Zealand (LINZ) [70]	
Regional Council and Territorial Authority boundaries	Statistics New Zealand [71, 72]	
Businesses, including food outlets	Zenbu [73]; Googleway R package [74]	Assessing the temporal validity of open source-business data; Gap filling council provided GIS data
Google Street View (GSV) images	Streetview python API [75]	

Historic data for the study period was requested from councils, however datasets were based on current district council data. While these were considered up-to-date at the time of provision, it was not known whether the environmental features in these datasets were present at the time the Whānau Pakari intervention was carried out (2012–2016). Furthermore, data coverage varied for each of the district councils within the Taranaki region. Some district councils were unable to provide all requested data, resulting in gaps in the overall dataset. Table 3 outlines data coverage for each district council region in the study area, and shows that data coverage was incomplete for public transportation, pedestrian crossings, on-street parking, traffic calming features, parks and playgrounds, water fountains, and registers of businesses. Parks and playgrounds in Aotearoa are generally council-managed recreational reserves rather than natural spaces, open spaces, or blue spaces.

**Table 3:** Data provided by district councils in the Taranaki region

Data	New Plymouth	Stratford	South Taranaki
Land use zoning	✓	✓	✓
Footpaths	✓	✓	✗
Cycleways	✓	✓	✓
Public transportation	✓	✗	✗
Streetlights	✓	✓	✓
Pedestrian crossings	✓	✓	✗
On-street parking	✓	✓	✗
Traffic calming features	✓	✗	✗
Parks and playgrounds	✓	✗	✓
Water fountains	✓	✗	✗
Register of businesses	✗	✗	✓

✓ denotes that data was available; ✗ indicates the data was unavailable

To fill these gaps in district council datasets and review the temporal validity of provided data three key objectives were decided upon:

1. Fill gaps in data in the Stratford and South Taranaki District Council regions (see Table 3) (*n* = 41 activity spaces).
2. Validate the temporal validity of public transportation, speed bump, and pedestrian crossing data provided by New Plymouth District Council (see Table 3) (*n* = 826 data points).
3. Validate the temporal validity of open-source data on food outlets (see Table 2) (*n* = 1353 data points). While food outlets data were not ‘date-stamped’, they were selected as open-source data to be validated because they are the most likely of the five open-source datasets to regularly change. The locations of schools, and road centrelines are relatively stable, while regional council and territorial authority boundaries are administrative constructs unable to be validated with GSV.

To achieve these objectives, a practical method using GSV images was developed. We found GSV coverage for all public roads in Aotearoa, including the Taranaki region, and image capture began in 2008. Therefore, it was possible to download historic, date-stamped, images. Furthermore, the street-level angle of the images allowed many features of interest (e.g., bus stops) to be identified. While GSV images can be downloaded for most coordinates that lie along the road network, it was not feasible to download and review GSV images for every section of road in the entire study region as this would have resulted in more than 490,000 images. Furthermore, the goals of the wider project were to investigate the relationship between environmental features and the health, dietary behaviours, and physical activity behaviours of children who participated in the Whānau Pakari trial. Therefore, it was decided to narrow down the area for which we would ‘gap-fill’ council data to activity spaces in the Stratford and South Taranaki District Council Regions. Before commencing analysis, all GIS datasets were converted to the NZTM2000 Mercator projection [76] for consistency. NZTM2000 is commonly used for small scale mapping in Aotearoa and it has a unit of metres, allowing for the meaningful mapping of distances.

### Stage 2: activity spaces and coordinates

**Fig. 3:**
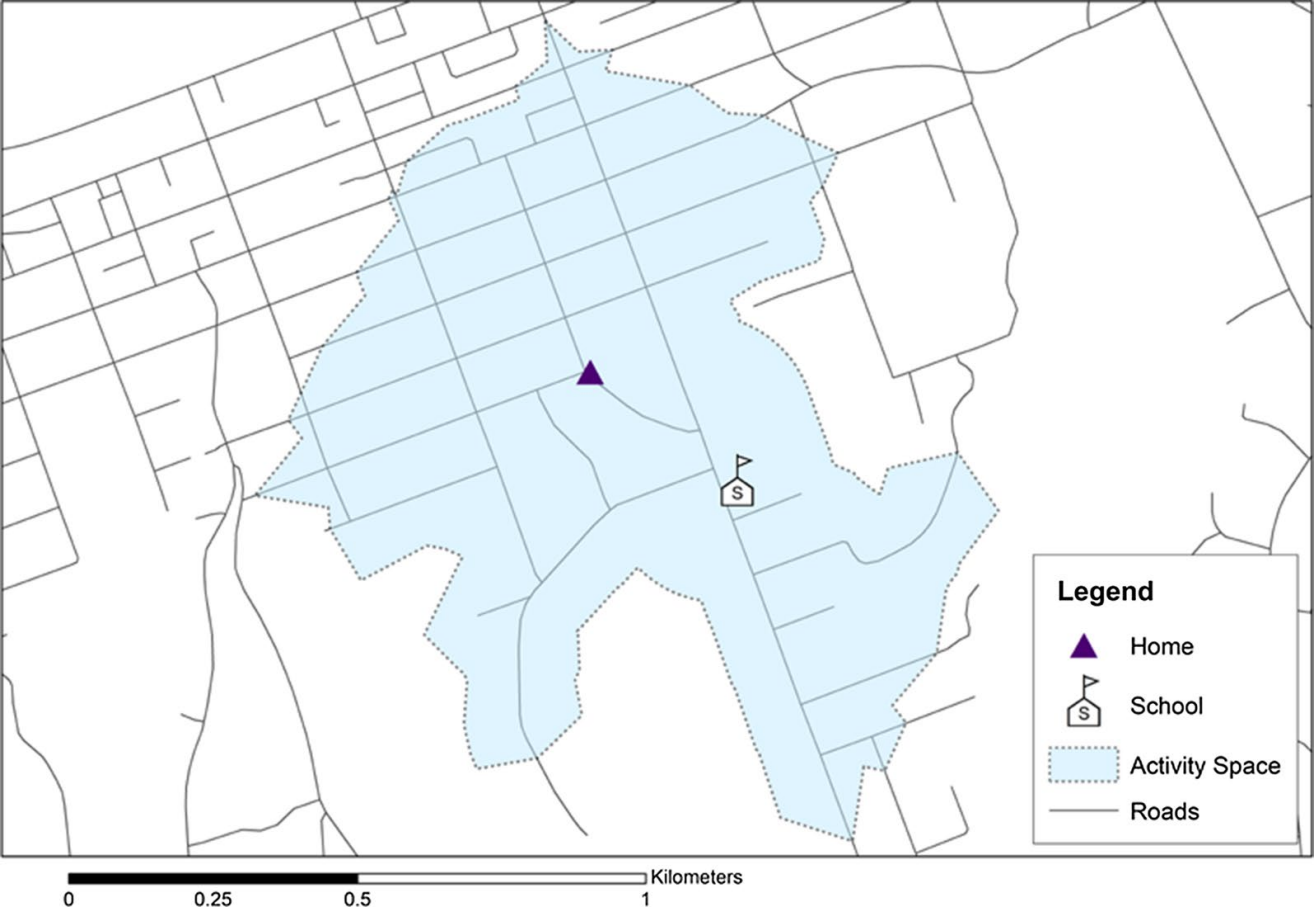
A hypothetical activity space

In this study, child-specific activity spaces were created to estimate the environments that individual children in the Whānau Pakari study were potentially exposed to. Common locations where children spend time are likely to include the area around their home and school, and the route between these two settings. In light of the previous literature in this area outlined in the introduction, individual child-specific activity spaces were developed for each participant in the KYNS study using ArcGIS 10.7.1. Participant residential addresses were geocoded and an 800 m road network buffer around children’s home addresses and their nearest school was created. A 200 m buffer around the estimated route between home and school was also created and included in the activity space to link the home and school environment. Figure 3 shows a hypothetical activity space.

### Stage 3: deriving coordinates and accessing GSV images

Once activity spaces had been created, they were used to define the area in the Stratford and South Taranaki districts within which missing data would be collected using GSV images. There were 41 activity spaces within the study area, and these spaces contained more than 200,000 m of road network. Points at 35-m intervals were generated along the road network within each activity space. A sensitivity analysis was carried out on one activity space to determine the appropriate distance between points along the road network that would both ensure comprehensive coverage of each activity space, while also minimising the total number of GSV images. Distances of 10, 20 30 40 and 50 m between points on the road network (and corresponding coordinates) were tested. Distances of 10 m and 20 m resulted in a high number of ‘duplicate’ or very similar GSV images being downloaded. Conversely, distances of 40 m and 50 m between points resulted in gaps and meant that features could not always be seen in consecutive GSV images, and therefore there could be some features that were missed. Thirty-meter distances provided good coverage of the test area, but also resulted in many images being downloaded and still produced many duplicates. As a result, a distance of 35 m was tested and determined to be the most appropriate for the purposes of this study. Thirty-five metres was used as this distance reduces the total number of images that need to be downloaded and manually reviewed, while ensuring that all portions of a street and all features of the environment are captured in the GSV images that are downloaded. In total, 6593 points were created. The coordinates of each point (using the WGS1984 coordinate system) were then derived and used to download corresponding GSV images for the period 2012–2016.

To assess the temporal validity of the data provided by New Plymouth District Council (NPDC), the locations of all ‘point’ data, including public transportation, pedestrian crossings, and speed bumps in the NPDC region were converted to WGS1984 coordinates. In total 826 coordinates were derived and were then used to download corresponding GSV images for the period 2012–2016 (the period of the Whānau Pakari trial).

To address objective (3), the locations of food outlets across the Taranaki region were identified using two open-source approaches. The freely available ‘Zenbu’ database lists businesses in Aotearoa, and all food businesses in the Taranaki region were extracted and geocoded. The second approach used the ‘Google Places’ function in the R ‘googleway’ API package [74]. The googleway package allows users to query Google Maps for information on a variety of categories including type establishments, a geographic location and a search radius. To create a list of food outlets in the Taranaki region, a search radius of 30 km was applied to 19 locations across the region, and the following place tags relating to food outlets were used: bakery, café, convenience store, gas station, liquor store, meal takeaway, park, restaurant, shopping mall, and supermarket. A CSV file of outlet names, coordinates, and type of establishment was produced, cleaned and duplicates were removed. Finally, the Zenbu and Google datasets were combined, and any remaining duplicate records were removed. The coordinates of each listed food outlet were then used to download GSV images for each location to validate both the spatial and temporal accuracy of this open-source data.

While current and historic GSV images can be manually downloaded from Google Maps, it is more efficient to automate this process when accessing thousands of images. The ‘streetview’ python package, originally developed by Letchford [75] was modified by Zhang [51], further adapted by this paper’s author JW, and then used to automatically download GSV images based on the coordinates derived from stage 2. For each coordinate four images with different compass headings were downloaded to give a 360º view of the location. The modified streetview package also allowed for images from specific years to be requested. GSV images taken during the period of the Whānau Pakari trial (2012–2016) were considered ‘in range’ while images taken outside these dates were filtered out and not downloaded. Finally, the package also created a CSV file which recorded key information associated with each downloaded image. This included an image ID (the same as the ID of the coordinates it is associated with), the coordinates from which the image was taken, and the month and year that the image was taken. In total 28,078 GSV images were downloaded in stage 3.

### Stage 4: coding environmental features from GSV images

Three research assistants (RAs) were trained to code GSV images and provided with a detailed set of instructions (developed based on Egli’s [77] data collection protocol), a data dictionary, training images, and a checklist in the form of an excel spreadsheet to record data into. Environmental features of interest were features that had not been provided in one or more district council dataset, features that could be readily identifiable in GSV images, as well as food outlets and physical activity features. These included: footpaths, on street parking, pedestrian crossings, speed bumps, traffic calming signs, public transportation, food outlets—including ‘unexpected sources of food and drink’ such as petrol stations [78], and physical activity features such as parks, playgrounds, and sports facilities. During formal data collection RAs were provided with batches of GSV images and worked individually to code the features. When RAs completed each batch of images, any difficulties that arose with coding was discussed with JW and ND and instructions were further refined. If the main function of a business was unclear, JW clarified this by searching the locations using Google to find more information. A randomly selected subset of 10% of images were reviewed by JW to ensure that features were continuing to be accurately identified and coded by all RAs. If errors had occurred, then retraining was conducted as needed. If no errors were found, then RAs were provided with the next batch of images.

### Stage 5: geospatial data and descriptive statistics

Each excel sheet of results was merged into a single CSV file which included the coordinates of each GSV image, and any environmental features recorded at that location by the RAs. These coordinates were mapped in ArcGIS 10.7 using the ‘Display XY Data’ feature. A new shapefile for each feature was then created by selecting ‘points’ where that feature was marked as present. This process was straightforward for point data such as public transportation or food outlet locations. To create shapefiles for ‘line’ features such as footpaths, we first mapped all coordinates with ‘footpaths’ as a tagged feature. We then calculated a 20 m buffer around these points to recognise that footpaths are not located at a single point, and that it is reasonable to expect that a footpath is visible 20 m from the location that a GSV image was captured. 20 m was used rather than 35 m because neighbouring coordinates would still have overlapping buffers at this distance. The buffer layer was then used to ‘clip’ the road network layer, with the resulting shapefile representing sections of the road network within the 41 selected activity spaces that also have a footpath. Once shapefiles of features identified using GSV were created, the ‘merge’ function was used to combine each ‘new’ shapefile with a copy of the original council-provided-data to create a gap-filled dataset. Shapefiles of validated selected New Plymouth District Council data and food outlets were considered to represent temporally accurate versions of the original datasets, and were not merged with the original data.

Simple descriptive statistics were calculated to outline how many novel features were identified in the 41 activity spaces, as well as the number of NPDC provided features and food outlets that could be temporally validated using GSV images.

## Results

Although GSV currently provides coverage for all public roads in Aotearoa, there may have been gaps in early versions of the service. Therefore, GSV images may not always be available for specific years. Overall, of the 34,168 images requested using the Google API using our modified python package, 28,078 (82%) of the returned images were taken between 2012 and 2016, while 6090 (18%) were out-of-range. Research assistants took a combined 98 h to review in range images and identify environmental features of interest. We mapped the locations of both GSV image requests and out-of-range images that were returned and examined their spatial distribution. 

**Fig. 4:**
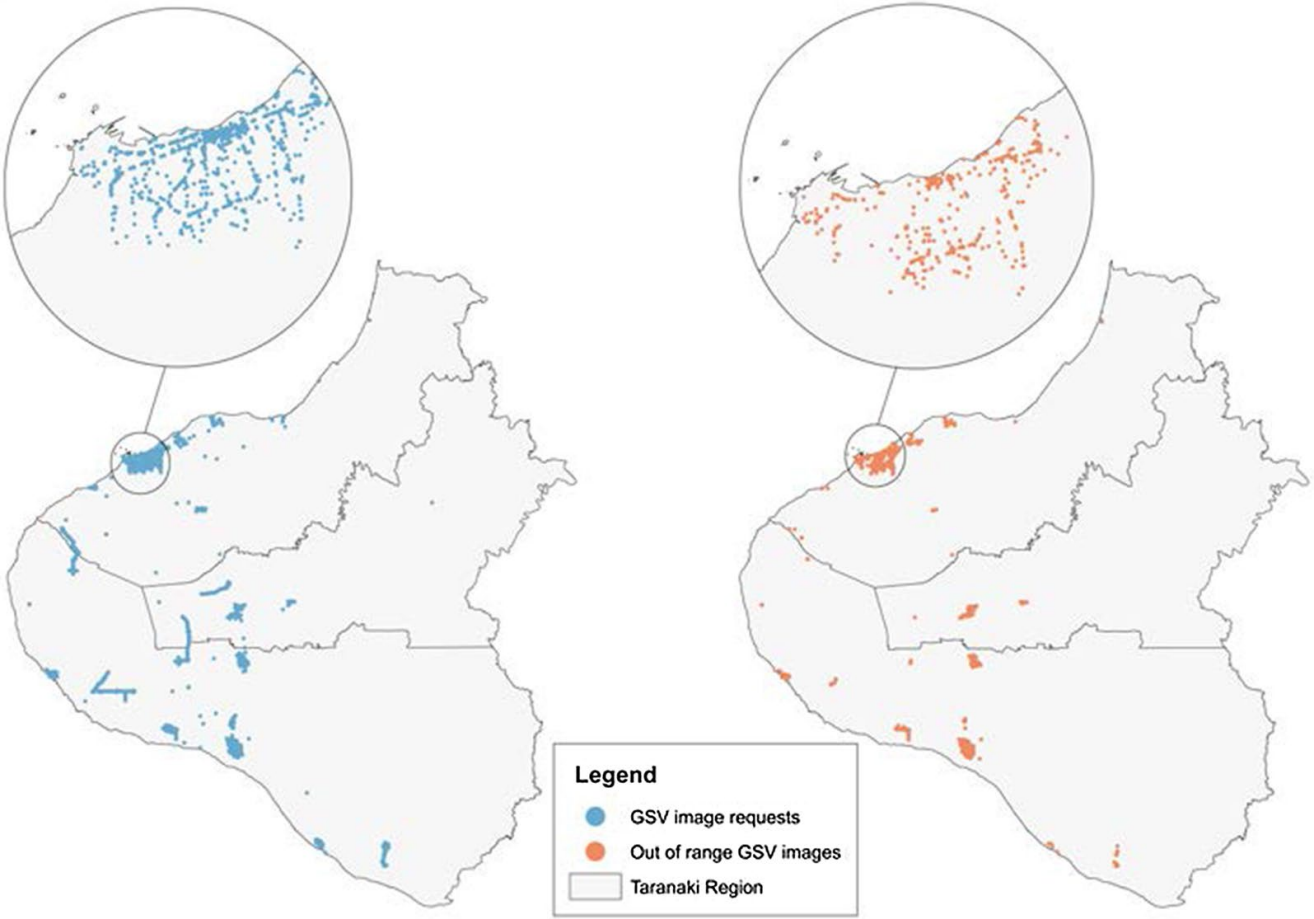
The spatial distribution of all requested GSV images, and locations where ‘out-of-range’ results were returned

Figure 4 displays the locations of all requested images and highlights the locations where an ‘out-of-range’ image was returned. Figure 4 indicates that out-of-range images are distributed evenly across the region and do not appear to be skewed towards any particular area.

Of the 26,372 requests for GSV images in the Stratford and South Taranaki districts, 21,665 (82%) in range images were returned. A total of 5166 coordinates with environmental features of interest were identified across the 41 activity spaces, and are listed in Table 4.

**Table 4:** Environmental features of interest identified in activity spaces using an historic GSV approach

Feature	Coordinates identified (*n*)	% (row)
Footpath	3378	65.0%
Cycleways	9	0.2%
Public transport	21	0.4%
Pedestrian crossings	72	1.4%
On-street parking	1310	25.4%
Traffic calming features	230	4.5%
Parks, playgrounds, physical activity features	67	1.3%
Food outlets†	79	1.5%
Total	5166	100%

^‡^NB: This number only includes food outlets that were identified in activity spaces, and does not include the 644 food outlets validated using open-source data

Of the 3304 requests for images relating to New Plymouth district council provided data, 2264 (69%) were in range. From these images, RAs were able to validate the presence of 402 features, an overall validation rate of 49%. Table 5 describes the number and proportion of temporally validated NPDC data points.

**Table 5:** NPDC provided data validated using historic GSV images

NPDC Feature	Data points provided (n)	Data points validated (n)	Data points validated (%)
Public transport—Bus shelters	98	44	45
Public transport—Bus signs	410	179	44
Speed bumps	116	52	45
Pedestrian crossings	202	127	63
Total	826	402	49

**Fig. 5:**
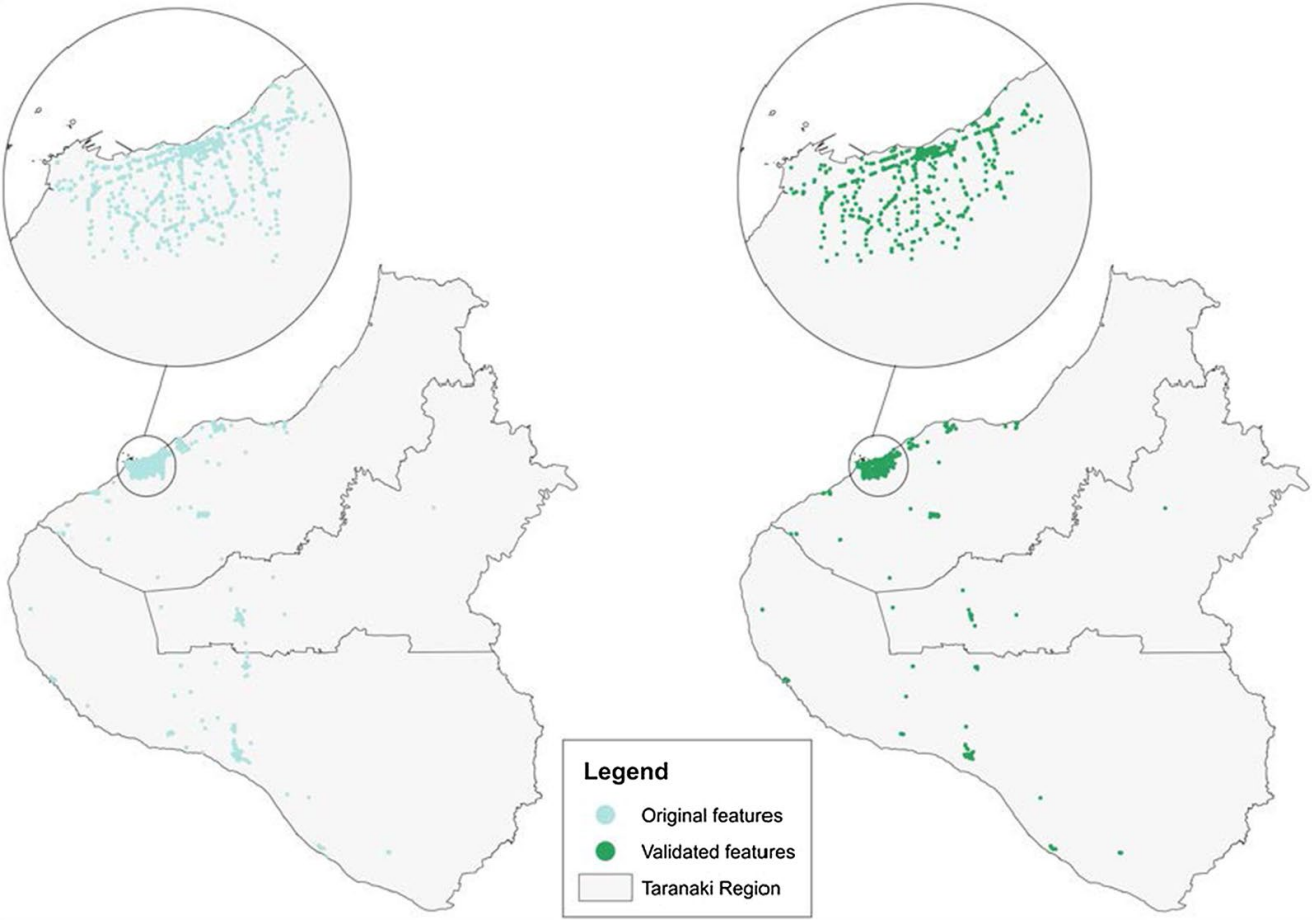
Food outlets and NPDC features that were temporally validated using historic GSV images

In total, 4492 requests for GSV images of 1123 potential food outlets (derived from Zenbu and Google Maps) were sent, returning 4149 (92%) in range images. RAs were able to validate the presence of 664 food outlets, an overall validation rate of 55%. Figure 5 shows food outlets and NPDC features that were identified and validated using historic GSV images.

## Discussion

The aim of this paper was to outline an approach to test the sensitivity and specificity of GIS datasets using GSV images. We have described an approach that can be applied to both ‘filling gaps’ and examining the temporal validity of GIS datasets. A substantial number of features were identified through this approach, highlighting the limitations of many GIS datasets. Researchers should not assume that secondary GIS datasets are complete and temporally valid.

The major finding of this study was that over 5000 locations were identified as having relevant features of interest that were not included in council datasets. These locations were within activity spaces only (a subset of the entire council regions) and therefore the total number of missing features in the council datasets is likely to be much larger. Another key finding was that 402 (49%) features in the New Plymouth District, and 664 (55%) food outlets across the Taranaki region were temporally validated using historic GSV images. While the GSV approach has resulted in a reduction in the total number of NPDC features and food outlets, it has improved the sensitivity and specificity of these datasets by only including features that were definitely present during the 2016–2016 study period. These validation rates are relatively low. While further investigation is needed, this suggest that secondary GIS datasets provided by organisations such as district councils or open-source repositories cannot be assumed to be complete or have temporal validity. Researchers should therefore consider triangulating a sample of their GIS datasets to estimate levels of completeness and accuracy. While the availability of GIS data were limited for the more rural councils (Stratford and South Taranaki) in the Taranaki region, our results displayed in Fig. 4 suggest that GSV image coverage was similar for both rural and urban areas, suggesting that our approach is likely to be applicable to other mixed urban–rural contexts in Aotearoa, and potentially internationally.

Despite the strengths of this approach, there are several limitations that should be noted. Firstly, it is difficult to determine how accurate the temporal validation of district council and open-source data were. This is because the absence of a GSV image of a feature from a particular year may be due to several reasons. One is that the feature was in fact not present at that date. Another is that even if the feature did exist no GSV image was available for that date. Finally, although RAs were trained comprehensively, it is possible that some features were missed. Ideally, our approach to validating GIS datasets with GSV images should be empirically tested with date-stamped data. The 4-year study period that we used as a date range for downloading GSV images of the study area is quite broad, meaning that built environments could change *during* this timeframe as councils update their infrastructure. Researchers should be aware of this limitation, and future work should examine how environments change within study periods. Where coverage is good and images are frequently updated, this could also involve using GSV to monitor changes in advertising environments, the impacts of rapid urbanisation and housing intensification, and the impact of specific policies targeting built environments. A further limitation is that some features are not suited for detection using GSV images. Smaller features such as water fountains within parks or playgrounds are unlikely to be visible in GSV images which are taken from the road. It is important to note that this research has focussed on using GSV to identify and validate environmental features of relevance that are discussed in the research literature. This has not included features or locations of importance for the health and wellbeing of indigenous children, such as marae or landmarks of cultural significance. We recognise that this is an important gap and intend to address it in future research.

While GSV has previously been used to assess environments in health research and is a promising tool for conducting street audits [47], there are issues and limitations that researchers should be aware of. The availability of GSV images varies globally, with better coverage in the ‘Global North’ and no availability in large parts of Africa, South America, the Middle East, India, China, South East Asia, and Russia [44, 79]. These gaps in coverage are due to a range of political, economic, legal, and technical factors [45]. Information around frequency of collection is not made publicly available by Google, and their website only contains information about the equipment used to capture images, areas that are currently covered, and the areas they are currently imaging [79]. Research also suggests that image availability and the frequency of capture also varies within cities, with wealthier neighbourhoods having higher rates of image availability, and more recently captured images [44]. The spatio-temporal instability of GSV imagery dates has also been critiqued as a weakness of using GSV to systematically observe the built environment [46]. The desktop version of GSV automatically shows the most recently captured image for a location, and this capture date can vary, making it difficult to meaningfully compare locations. The approach that we have outlined overcomes this issue to some degree. By setting a date range of interest within which images will be downloaded, GSV can be ‘forced’ to provide more consistent images. Furthermore, researchers using our approach are able to determine their own temporal sensitivity thresholds by setting a date range that includes a single month, or extending the period of interest to an entire decade. While not all locations will have images available within each specified date range, our approach offers more flexibility in addressing issues of the spatio-temporal instability of desktop GSV imagery. While the recent inclusion of user-uploaded data, including images, into google maps could add variation to the quality and veracity of images, this user-uploaded data is incorporated as standalone unlinked “Photo Spheres” which are subject to acceptance criteria [45]. Furthermore, our described method only allows for the downloading of images captured by Google, which include a “Pano ID”, thereby excluding user-uploaded content. Our approach has potential for the validation of the temporal accuracy and overall completeness of open-source data, such as Open Street Map or Ordinance Survey POI data that can be downloaded for specific dates to align with study data. Provided that Google has captured an image in this same date range, our approach allows archived GSV images to be downloaded for the same specific dates. Our approach could therefore be one way to validate the accuracy and completeness of such open-source datasets, or secondary data that is provided with a timestamp.

This paper has outlined a practical approach for using GSV to supplement spatial and temporal information that is often missing from GIS datasets. Since GSV images have spatial coordinates associated with them, and can be accessed through an API, this is a potentially important approach for researchers to test and improve secondary GIS datasets. Although GSV images can be automatically downloaded, the process we have described is still reliant on manual analysis of environmental features by RAs meaning that it would be time consuming for larger projects, and potentially prone to human error [80]. While our approach is currently practical for projects and studies undertaken in a small geographic area, there is potential for scalability and replication of this approach with different datasets and different spatial contexts, or for use validating a subset of spatial data to gauge the estimated completeness and temporal validity of an overall dataset. Furthermore, researchers have also demonstrated how feature identification could be automated [51, 56], and advances in machine learning and AI are likely to lead to future improvements in this area. Machine learning technology could be employed to further automate the process of validating, and filling gaps in GIS datasets. This could improve the efficiency and accuracy of stages 4 and 5 of our methodology, allowing for larger scale analyses to be undertaken. This could involve larger study areas, more in-depth analyses of multiple environmental features, and potentially longitudinal examinations in changes to the built environment. The development of an entirely automated tool to download historic GSV images and harness machine learning to classify and geocode features of the built environment was beyond the scope of our current project, but would be very useful for assessing the accuracy and completeness of secondary GIS datasets. The original streetview python script has been made freely available for download from GitHub (https://github.com/robolyst/streetview) and our modified version is available on request.

## Conclusion

Geospatial datasets are not always complete and may not include information on the temporal specificity of data. While this poses a significant limitation to retrospective and longitudinal studies examining the relationships between built environments and health outcomes, we have developed a practical approach for addressing these limitations. GSV images can be utilised to improve secondary GIS data.

## Data Availability

The datasets generated during this study are not publicly available because they may potentially compromise the identity of children who participated in the Whānau Pakari study. Some of the datasets analysed in this study are open-source and are available from the links listed in the reference section.

## Abbreviations

API: Application Programming Interface
GIS: Geographic information systems
GPS: Global Positioning System
GSV: Google Street View
KYNS: Knowing Your Neighbourhood Study
LINZ: Land Information New Zealand
NPDC: New Plymouth District council
OSM: Open Street Map
RA: Research assistant
SDC: Stratford District Council
STDC: South Taranaki District Council

## Footnotes

1 New Plymouth District Council have a ‘GeoHUB’ with GIS data is freely available online, which could be considered ‘open-source data’. However accessing similar data from Stratford and South Taranaki District Councils required making direct requests to the GIS teams at these councils. For consistency, data from NPDC has been grouped together with other councils and is considered ‘council data’.
